# Management of relapsing *Plasmodium vivax* malaria

**DOI:** 10.1080/14787210.2016.1220304

**Published:** 2016-08-31

**Authors:** Cindy S Chu, Nicholas J White

**Affiliations:** ^a^ Shoklo Malaria Research Unit, Mahidol–Oxford Tropical Medicine Research Unit, Faculty of Tropical Medicine, Mahidol University, Mae Sot, Thailand; ^b^ Mahidol–Oxford Tropical Medicine Research Unit, Faculty of Tropical Medicine, Mahidol University, Bangkok, Thailand; ^c^ Centre for Tropical Medicine and Global Health, Nuffield Department of Medicine, University of Oxford, Oxford, UK

**Keywords:** *Plasmodium vivax*, primaquine, glucose-6-phosphate dehydrogenase deficiency, review, *Plasmodium vivax* relapse, 8-aminoquinoline, *Plasmodium vivax* treatment, radical cure, anti-malarial efficacy, chloroquine

## Abstract

**Introduction:** Relapses are important contributors to illness and morbidity in *Plasmodium vivax* and *P. ovale* infections. Relapse prevention (radical cure) with primaquine is required for optimal management, control and ultimately elimination of *Plasmodium vivax* malaria. A review was conducted with publications in English, French, Portuguese and Spanish using the search terms ‘*P. vivax’* and ‘relapse’.

**Areas covered:** Hypnozoites causing relapses may be activated weeks or months after initial infection. Incidence and temporal patterns of relapse varies geographically. Relapses derive from parasites either genetically similar or different from the primary infection indicating that some derive from previous infections. Malaria illness itself may activate relapse. Primaquine is the only widely available treatment for radical cure. However, it is often not given because of uncertainty over the risks of primaquine induced haemolysis when G6PD deficiency testing is unavailable. Recommended dosing of primaquine for radical cure in East Asia and Oceania is 0.5 mg base/kg/day and elsewhere is 0.25 mg base/kg/day. Alternative treatments are under investigation.

**Expert commentary:** Geographic heterogeneity in relapse patterns and chloroquine susceptibility of P. vivax, and G6PD deficiency epidemiology mean that radical treatment should be given much more than it is today. G6PD testing should be made widely available so primaquine can be given more safely.

## 1. Geographical distribution of *Plasmodium vivax* malaria

**Figure 1.**
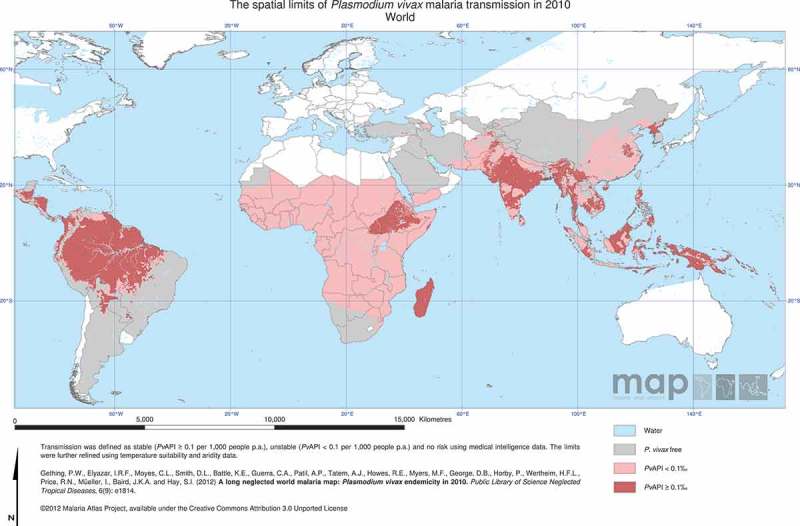
Estimates of the global distribution of P. vivax. API = (confirmed cases during 1 year/population under surveillance) x 1000. Although P. vivax is shown as occurring across Africa, it is rare west of Uganda in Southern and Eastern Africa, and south of Mauritania and Mali in West Africa.

Areas endemic for *P. vivax* cover 95 countries comprising approximately 44 million square kilometers on five continents. Approximately, 48% of the world’s population or 2.48 billion persons are potentially at risk for *P. vivax* infection (most parts of China and large areas of India are at very low risk) [1,2]. The 35 countries in Asia have high-population densities and represent over 80% of the global population at risk [3]. In the Asia- Pacific region, approximately 9% of the global population is potentially at risk. With an annual parasite index per 1000 persons per year for *P. vivax* (PvAPI) of ≥7, high transmission of *P. vivax* occurs in parts of India and parts of South-East Asia (Myanmar, Indonesia), and transmission intensities are highest on the island of New Guinea [2] (Figure 1). In contrast, the Americas have lower population densities, thus despite having areas of high transmission (in the Amazon region of Brazil PvAPI is >100 and in western Colombia overall API is >35 with 60% of malaria cases caused by *P. vivax*), and contributing approximately 22% of the endemic global land area they represent only 5.5% of the global population at risk [3–5]. The regions of Africa (notably the horn of Africa and parts of West Africa) and the Arabian Peninsula are less well known for being endemic for *P. vivax* malaria and represent 3.5% of the global population at risk [6]. There are over 60 potential anopheline vector species in Asia and Asia Pacific region, 20 potential vectors in the Americas and 10 in the African regions [6]. *P. vivax* sporogony occurs at ambient temperatures as low as 16°C so this parasite is found over a greater geographic range than other human malarias.

## 2. The biology of *P. vivax* malaria relapse

### 2.1. Early observations on relapse

**Figure 2.**
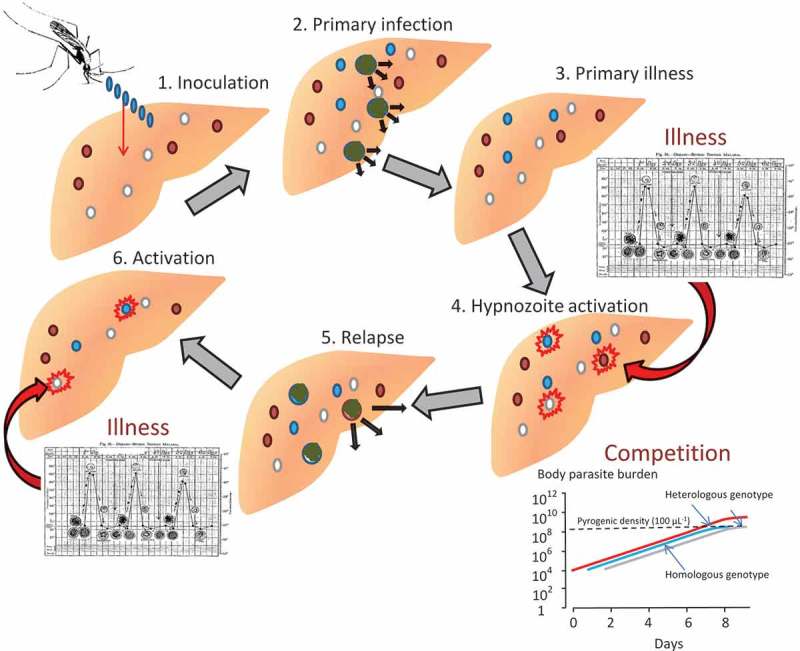
Proposed mechanism and sequence of *Plasmodium vivax* relapse activation in a malaria endemic area. In the example at the time of infection the individual already has hypnozoites of two different genotypes acquired from two previous inoculations which are latent in the liver (red and white circles). Half the newly acquired infection sporozoites (blue) develop into pre-erythrocytic schizonts and half become dormant as hypnozoites (blue circles) (this is the estimated proportions for tropical ‘strains’). Illness associated with the blood stage infection activates a low fraction of the hypnozoites. In this example one of each genotypes develops into preerythrocytic schizonts. By chance the progeny of one of the preexisting latent hypnozoites reach pyrogenic densities before the progeny of the recently inoculated hypnozoite (inset: ‘competition’). The consequent illness then suppresses further multiplication of the blood stage infection so that the progeny of the other two prerythrocytic schizonts may not reach transmissible densities. The ensuing illness activates some of the remaining hypnozoites (the same fraction as were activated initially) and relapses continue until either the number of hypnozoites is exhausted or some fail to be activated. If there are some hypnozoites which fail to be activated these may be activated by a subsequent malaria infection. Figure available (open access): Nicholas J. White. Determinants of relapse periodicity in *Plasmodium vivax* malaria. Malar J. 2011;10(1):297. (Full color available online)

In contrast to malarias caused by *P*. *falciparum, P. malariae,* and *P. knowlesi*, both *P. vivax* and *P. ovale* form latent liver stages (hypnozoites) that remain dormant for weeks or months before awakening to produces relapses of malaria (Figure 2). Patients who are cured of their blood stage infection may, therefore, have a subsequent infection without reinfection. The presence of this latent (exo-erythrocytic) stage had been hypothesized as early as 1893 [7] and was supported by large epidemiologic studies performed in the Netherlands (where relapse intervals or incubation periods of 8–9 months were usual) [8]. Manson provided proof in 1901 when relapse was documented 9 months after mosquito induced *P. vivax* malaria in a human volunteer (his son!) living in a non-endemic setting [9]. During the era of malaria therapy, it was well recognized that relapses followed mosquito transmitted but not blood-transmitted vivax malaria [10,11]. Heavy inoculations (large numbers of mosquito bites) were noted to increase the number of relapses [12] and shorten the interval between the primary infection and the first relapse [13]. Acquired immunity reduced the probability of symptomatic relapse; when artificially induced *P. vivax* infections were allowed to run a protracted course the probability of relapse was reduced, and the patient could not be later reinfected with the same strain [14]. However, if prompt antimalarial treatment was given, attenuating the acquisition of immunity, then symptomatic relapses were more likely [15–17]. For most of the first half of the twentieth century, long-latency *P. vivax* (where either primary infection or first relapse occurred months after the initial mosquito bite) was the main *P. vivax* phenotype characterized. The short-latency form was documented by Sinton and his colleagues in soldiers in Kasauli, Himachal Pradesh, India, but was not well recognized elsewhere until large numbers of soldiers fighting in the Indo-Burman and South Pacific theatres during the Second World War experienced multiple short interval relapses. In contrast to the long-latency *P. vivax*, relapses with short-latency infections (Chesson strain) occurred earlier and at higher rates, with relapse rates as high as 90% within 6 weeks of the treated primary infection [18,19].

### 2.2. Geographical distribution of relapse latency

In North America, Europe, and Russia before elimination, the prevalent *P. vivax* was of the long-latency form, with either an approximate 9-month incubation period or with a 2-week incubation period for the primary infection and then a relapse interval of approximately 9 months (typified by the Madagascar and St Elizabeth strains used extensively in malaria therapy). In contrast in South-East Asia and Oceania, *P. vivax* is of the short-latency form where relapses occur approximately 3 weeks after rapidly eliminated antimalarials (artesunate, quinine) and 6–7 weeks after slowly eliminated drugs, such as mepacrine, chloroquine, mefloquine, or piperaquine. This was characterized by the Chesson strain evaluated extensively in volunteer studies. In tropical regions, the frequent relapse form predominates, although long-latency forms may coexist. Relapses from the Chesson strain could still occur after very long intervals. Intermediate latency (time to relapse 3–6 months) has also been described [20], but it is unclear whether this represents a distinct entity.

### 2.3. Hypnozoite activation

In 1982, small forms of *P. vivax*, considered to be hypnozoites, were seen on immunofluorescence microscopy within liver cells of *P*. *vivax*-infected chimpanzees [21]. Over 30 years later, little is known about the biology of hypnozoites and the determinants of latency. The relapse patterns in *P*. *vivax*-infected patients in non-endemic settings [13], the remarkable periodicity of relapse, the observation that relapses are often caused by a genotype different to that causing the primary infection, and the very high rates (~30% in South-East Asia) of *P. vivax* occurring after *P. falciparum* infections [22], have led to the theory that hypnozoites can be activated by external stimuli, such as a malaria infection [17] or other infectious disease [23,24]. In endemic settings, recrudescence, reinfection, and relapse (collectively known as recurrence) cannot be distinguished reliably because relapses in endemic areas are commonly caused by heterologous genotypes [25]. However, in infants first recurrences after the first *P. vivax* infection in life are usually genetically homologous [26]. These observations have led to the suggestion that residents in vivax endemic areas accumulate a ‘bank’ of hypnozoites in the liver from repeated sporozoite inoculations which can be activated by malaria or other serious infections [17].

## 3. Burden of *P.* *vivax* infection

In South-East Asia entomological inoculation rates for *P. falciparum* and *P. vivax* are often similar but the age-related incidence of malaria is very different. *P. falciparum* incidence is highest in young adults whereas children less than 5 years old have higher rates of *P. vivax* infection than older children and adults [27–30]. This difference in clinical epidemiology is explained by relapse. A single *P. vivax* inoculation may cause multiple relapses. In contrast in areas where relapse is less frequent (i.e. India ~20%, South America ~30%) vivax malaria is reported more in older age groups [31]. As with *P. falciparum* infections, pregnant women, especially primigravidae have higher rates of *P. vivax* infection [32]; this increased risk also extends to postpartum women [33]. *P. vivax* also causes abortion and reduced birth weight [34–36]. In Southern Papua, where transmission is high, similar proportions of infants are hospitalized with *P. falciparum* and *P. vivax*. Anemia is the main clinical manifestation, and mortality rates from severe anemia in falciparum and vivax malaria are similar [37,38]. Repeated vivax malaria interferes with growth, development, and school performance, and in adults it results in loss of income and absence from work [39]. As the number of relapses or new infections increase, the clinical and economic burden of *P. vivax* infection also increases.

## 4. Morbidity associated with *P.* *vivax* infection

### 4.1. Malnutrition

In areas of higher transmission, *P. vivax* infections are associated with increased rates of underweight, wasting and stunting in children [31,38,40]. Larger reductions in growth velocity (nearly 0.1 cm per episode) in children from the Peruvian Amazon occurred with *P. vivax* malaria than diarrhea [41], suggesting that repeated relapses may have long-term chronic adverse effects on children. Children with pre-existing malnutrition are at higher risk for mortality from both *P. falciparum* and *P. vivax* malaria [30].

### 4.2. Anemia

Frequent relapses of short-latency *P. vivax* infections cause anemia [42,43]. In Papua, Indonesia (high-transmission, frequent relapse), anemia in young infants (<3 months) hospitalized with *P. vivax* malaria was more profound than in *P. falciparum*. The prevalence of severe anemia was greatest in the >1– 3-month age group [38]. In Turbo, Colombia (high-transmission less frequent relapse), prevalence of anemia was highest in children ≤6 years old [44]. Adult females with blood loss from menstruation and pregnant women with increased iron needs are also at particular risk for *P*. *vivax*-associated anemia [32,42].

### 4.3. Pregnancy and the perinatal period

In a review of 13 studies conducted in the Asia-Pacific region, where both *P. falciparum* and *P. vivax* are endemic, *P. vivax* was estimated to have caused 28.5% (range 5–100%) of malaria infections detected at antenatal clinics [45]. In India [46], Nepal [47], north-western Thailand [35], and Papua New Guinea [34] approximately 70–77% of women had malaria-related anemia at some point during pregnancy; in 1–19% of women, it was severe. Severe anemia was associated with premature labor, stillbirth, and low birth weight [45,48]. In Ethiopia, a cross-sectional community-based study showed that 90% of healthy pregnant women with asymptomatic parasitemia (48% *P. vivax*) were anemic (hematocrit <33%) [49]. Independent of anemia, even asymptomatic *P. vivax* infections in later pregnancy increase the risk of premature labor. When early detection and treatment were provided in an endemic area along the Thailand–Myanmar border, the risks of premature delivery or stillbirth were reduced by interrupting newly acquired infections at an early stage [32]. Symptomatic maternal *P. vivax* infections are a risk factor for premature labor and infant death (1–3 months of age) [35].

### 4.4. Other comorbidities

The association between symptomatic *P. vivax* malaria infections and concomitant bacterial infections has long been recognized [24]. In a recent study concomitant bacteremia was more likely during *P. vivax* infections in an underserved Indian population, and of the positive blood cultures, 5/6 grew Gram-negative bacteria including *Salmonella Typhi* and *Salmonella paratyphi A* [50]. Recent literature suggests that *P. vivax* can cause severe disease [30,37,51–57] but, as with falciparum malaria in high transmission settings, association does not necessarily imply causation. Other factors may also contribute to disease severity. Case reports and retrospective studies on severe *P. vivax* infection and associated clinical syndromes have often not collected data systematically on pre-existing chronic diseases and concomitant infections or performed investigations to diagnose or exclude them [58]. An extensive review of publications on severe *P. vivax* malaria from Brazil showed that 79% did not report the presence or absence of a comorbidity [59]. The presence of concomitant illnesses or chronic disease may contribute to the severity of disease; both Peruvian [60] and Indian patients who died [61] were older than survivors, suggesting that chronic or underlying diseases may complicate *P. vivax* infections. More recent studies on severe *P. vivax* malaria have included confirmation of *P. vivax* mono-infection with polymerase chain reaction (PCR) methods (although the greater sensitivity of PCR means that a greater proportion of parasite carriers will be identified) [62–65]. Case fatality rates vary by country with the lowest rates reported from Colombia (0.012%) and highest from Indonesia (0.063%) [66]. In areas where *P. vivax* is endemic, low-parasite densities are common in apparently healthy people so when they become ill a causative role is often ascribed to *P. vivax* where in truth the relationship is coincidental. False attribution of illness to *P. vivax* may also occur if the illness leads to activation of latent hypnozoites causing relapse [24]. Prospective trials with strict case definitions and supporting laboratory testing are needed to establish the true incidence of severe vivax malaria.

## 5. Contribution of vivax relapse to overall infection rate

The contribution of relapse to the overall burden of vivax malaria can be assessed by comparing recurrence rates between patients who take and do not take radical treatment with primaquine. Parasite genotyping cannot convincingly differentiate relapse from reinfection or recrudescence. Thus, most published studies have measured overall recurrence rates (relapse, reinfection, and recrudescence). These assume that any difference in recurrence rate between treatments can be attributed to killing of hypnozoites (and that contributions from asexual stage and causal prophylactic activities are negligible). From the first chloroquine efficacy studies in American soldiers with *P. vivax* of Pacific origin conducted immediately after the Second World War relapse rates were found to be 70% within 120 days after return from endemic areas [67]. Similarly, 53 years later in north-western Thailand (low transmission), a 79% recurrence rate within 63 days was recorded after chloroquine monotherapy [68]. More recently, a recurrence rate of 78% within 1 year after artesunate monotherapy was reported in Indonesian soldiers, returning from a high-transmission area [69]. Prospective studies measuring the incidence of new *P. vivax* infections in healthy populations are rare. In Papua New Guinea, which has very high *P. vivax* transmission, healthy children <6 years were treated with artesunate plus primaquine, artesunate only or placebo, then followed for 10 months. Relapse was estimated to contribute approximately 50% to the overall number of *P. vivax* infections [70]. More recent estimates are higher (80%). Although relapse undoubtedly accounts for the majority of recurrences, the effective causal prophylactic activity of primaquine should be factored in the calculation of how many malaria recurrences are attributable to relapse. Longer follow up periods will describe more accurately the temporal burden of relapse. Currently, available data suggests that relapses contribute significantly to the overall burden of *P. vivax* infection.

## 6. Treatment of *P.* *v* *ivax* malaria (new infections, recrudescence, and relapse)

### 6.1. Blood stage infection

For nearly 60 years, chloroquine has been the first-line treatment against *P. vivax* [71]. Chloroquine is very slowly eliminated (estimated terminal half-life of 1 month). There is one major biologically active metabolite, desethylchloroquine. Metabolism is by the liver cytochrome P450 CYP2C8, CYP3A4, and CYP2D6 isoforms [72]. Tissue binding is extensive resulting in an enormous apparent volume of distribution [73,74]. Chloroquine drug levels are detectable in cord blood, neonatal blood, and infant urine after maternal ingestion within the last 4 weeks of pregnancy [75]. Chloroquine is absorbed reliably even in sick patients [76] and is distributed extensively in the tissues with highest concentrations found in liver followed by the spleen, heart, kidney, and brain [77,78]. Thus, the treatment, traditionally divided over 3 days, can be considered a loading dose. When used chronically in high doses for the treatment of autoimmune diseases, chloroquine accumulates in the body with detectable levels in the plasma and red blood cells for months or in the urine for as long as 5 years after discontinuing treatment [79]. The dose needed to treat sensitive *P. falciparum* infections was 25 mg base/kg. This dose provides therapeutic levels in whole blood (>100 ng/mL corresponding to >10 ng/mL in plasma) for at least 28 days in *P. vivax* infections [80]. Very low doses (total dose 5 mg base/kg) were effective in experimental vivax malaria but are not recommended [71]. Although chloroquine has a low safety margin (a single dose of 20 mg/kg is considered toxic), it is generally well tolerated at the standard dose of 25 mg base/kg divided over 3 days for *P. vivax* infection [81].

#### 6.1.1. Chloroquine resistance

**Figure 3.**
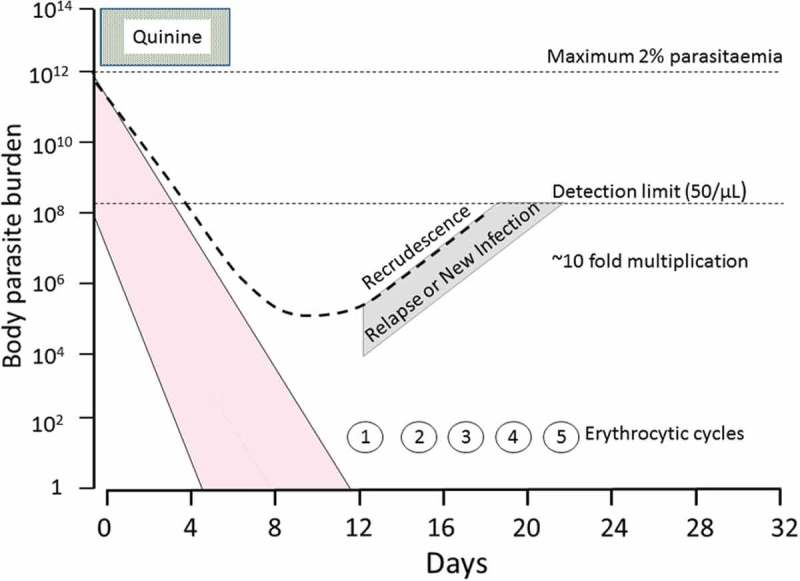
Pharmacodynamic responses in tropical vivax malaria. Parasitemias (shown in pink) in vivax malaria range up to 2% which corresponds to a total body burden of up to 10^12^ parasites in an adult. After treatment parasite densities decline by factors of between 10^3^ and 10^4^ per asexual cycle. In this example the treatment is quinine which is relatively rapidly eliminated so there is little post treatment suppression of multiplication. Approximately two weeks after the acute illness (and after one week’s intrahepatic development) the hepatic schizonts (range illustrated here is between one and ten) derived from activated hypnozoites burst to liberate merozoites. Multiplication is unrestrained and patent parasitaemias are reached approximately one week later. For a recrudescent infection (dotted line) to predominate over a relapse, asexual parasite killing must be reduced substantially. (Full color available online)

**Figure 4.**
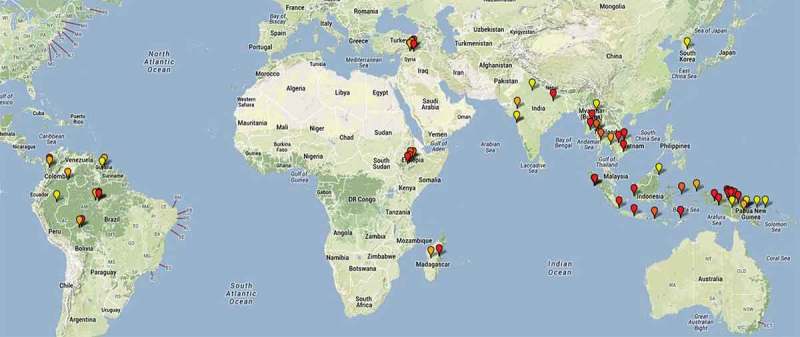
Geographic distribution of chloroquine resistant *P. vivax*. Yellow markers-case reports, Red markers->10% recurrence by day 28 (highly suggestive of resistance), Dark orange-recurrence is confirmed with chloroquine whole blood concentrations >100nM, Light orange->5% recurrence by day 28 (potential evidence of resistance). Map provided by personal communication with Professor Ric N Price. Full data available (open access): Price, Ric N., Lorenz von Seidlein, Neena Valecha, Francois Nosten, J. Kevin Baird, and Nicholas J. White. ‘Global extent of chloroquine-resistant *Plasmodium vivax*: a systematic review and meta-analysis.’ The Lancet Infectious Diseases 14, no. 10 (2014): 982–991.

Chloroquine resistance in *P. vivax* emerged decades after it did in *P. falciparum* despite enormous selection pressure from extensive use. Chloroquine resistance in *P. vivax* was first reported in Papua New Guinea in 1989 [82,83]. This was followed by case reports from Indonesia [84], Brazil [85], Myanmar [86], India [87], Guyana [88], Colombia [89], Peru [90], Cambodia [91], Ethiopia [92], Thailand [93,94], and Bolivia [95]. Several factors may have contributed to the slow emergence of chloroquine resistance, notably the relatively higher doses in terms of antimalarial activity, lower parasite burdens in vivax malaria, and the early relapses in infections with tropical ‘strains’, which pre-empt recrudescences and so outcompete *de* *novo* resistant infections [17]. Chloroquine resistance is usually described clinically as reappearance of patent asexual parasitemia within 28 days of a treatment dose in the presence of a therapeutic measured whole blood (>100 ng/mL) or plasma (>10 ng/mL) level of chloroquine. In tropical areas, the usual cause of recurrence with low level chloroquine resistance is a first relapse breaking through residual chloroquine levels and reaching patency within 28 days. Of course, this could also be a newly acquired infection or, if resistance is high grade, a recrudescence (Figure 3). Whichever the case the infection is likely to be chloroquine resistant if a full treatment course was taken. Although resistance can be confirmed by measuring blood chloroquine levels, this is not always done in chloroquine efficacy studies or case reports. The measurement of drug levels should be required before attributing clinical treatment failure to chloroquine resistance (Figure 4). *P. vivax* still remains sensitive to chloroquine in parts of South and East Asia with failure rates less than 5% in India [96,97], Pakistan [98], China bordering Myanmar [99], Vietnam [100], and Thailand bordering Cambodia [91,101,102]. In the Americas, studies show variable chloroquine efficacy depending on region; recent day 28 failure rates were 17.4% in Brazil [103] and 10.4% in northern Bolivia (9% of patients parasitemic at day 3) [95]. The day 28 failure rate in western Columbia was 7% (mean parasite clearance time 70 h) [89] and 0% in the east [104]. Failure rates (measured between 6.8% and 34% at 28 days) are rising in Thailand bordering Myanmar where an increasing proportion of patients have gametocytemia at presentation [101,105,106]. The highest rates of failure have been reported from Indonesia; in Papua where transmission is high 65% of chloroquine-treated children were parasitemic again within 28 days [107]. This has resulted in a change in national policy in Indonesia from chloroquine to artemisinin-based combination therapy (ACT) [108]. Chloroquine resistance may be confounded by inadequate dosing, especially in children [109] and overweight adults [110]. There is considerable heterogeneity among chloroquine efficacy studies that limits comparison of treatment failure rates and parasite clearance times [111]. In an effort to standardize study methodology for *P. vivax* therapeutic efficacy studies, study tools and a comprehensive map of studies assessing chloroquine resistance in *P. vivax* are available on the Worldwide Antimalarial Resistance Network website. (http://www.wwarn.org)

#### 6.1.2. Alternative treatments to chloroquine

*P. vivax* is generally sensitive to antimalarial drugs. Drugs evaluated include rifampicin [112], atovaquone–proguanil [113], artesunate [114–117], sulfadoxine–pyrimethamine [107,118,119], amodiaquine [120], and more recently ACTs: artesunate–amodiaquine [121], artemether–lumefantrine [119,122–124], dihydroartemisinin–piperaquine [69,91,93,119–122,125], and artesunate–pyronaridine [126]. In pregnant women, both amodiaquine and dihydroartemisinin–piperaquine are well tolerated and effective treatments [127,128]. Antifol resistance develops readily in *P. vivax* and in some areas sulfadoxine–pyrimethamine treatment is associated with high failure rates and slow parasite clearance times so this drug alone is not recommended for vivax malaria. Atovaquone–proguanil provides high cure rates but parasite clearance is relatively slow, and the drug is expensive. Artemisinins as monotherapies and ACTs provide rapid parasite clearance, but recurrence rates with artemisinin monotherapies by day 28 are high as these rapidly eliminated compounds do not prevent the first relapse. Antimalarials with longer elimination half lives (i.e. chloroquine, mefloquine, or piperaquine) provide post treatment prophylaxis and are associated with lower early recurrence rates when compared to antimalarials with shorter half lives. For example with dihydroartemisinin–piperaquine early recurrence rates are lower than with artemether–lumefrantrine [122] (lumefantrine is more rapidly eliminated than piperaquine). If early reinfections (in high transmission areas) or relapses (in areas with short-latency relapse) are prevented, this provides longer disease-free intervals and also reduces transmission potential [17]. This suggests that partner drugs combined with an artemisinin derivative should preferably be slowly eliminated (i.e. provide extended post-treatment prophylaxis) if radical cure is not provided. Dihydroartemisinin–piperaquine, with a piperaquine terminal half-life of approximately 28 days, has emerged as the most efficacious treatment for vivax malaria with low day 28 failure rates and rapid parasite clearance times [129]. Artesunate–mefloquine with a mefloquine terminal half-life of ~20 days is also very effective [114,130].

### 6.2. Radical curative treatment with primaquine

The radical curative efficacy of the 8-aminoquinolines has been recognized for over 75 years [131]. Primaquine, the only 8-aminoquinoline now widely available, is active against the developing pre-erythrocytic forms (causal prophylactic activity), the hypnozoites (only *P. vivax* and *P. ovale*), the asexual blood stages (except *P. falciparum*), and gametocytes (notably *P. falciparum*) of all human malaria parasites. After oral administration, peak primaquine concentrations occur within 1–3 h [132]. The elimination half-life is 4–6 h [74]. Primaquine is a prodrug for a currently unidentified bioactive metabolite. Characterizing the biotransformation of primaquine has been difficult because many of the metabolites are unstable. Primaquine is metabolized by the liver cytochrome CYP P450 enzymes and monoamine oxidase-A. The CYP2D6 mediated pathway is considered responsible for the formation of the metabolites associated with both the antimalarial efficacy and the hemolytic effects of the drug [133,134]. The enantiomers of primaquine, (+)-(*S*)-primaquine and (–)-(*R*) primaquine, have different rates of metabolism by CYP2D6 [135], which may explain partially the different toxicities that have been observed previously between the two [136]. Research is being performed to identify and hopefully measure levels of the reactive metabolites or their reaction products [137,138].

#### 6.2.1. Primaquine interactions

Primaquine taken on an empty stomach causes abdominal pain, so it is usually administered after food and/or drink. The fed state and the ingestion of grapefruit juice (a CYP3A4 inhibitor) were found to increase the bioavailability of primaquine as compared to the fasting state [139]. Concomitant dosing of quinine or chloroquine with primaquine or its predecessor plasmoquine (pamaquine) was shown to increase anti-relapse activity as compared to sequential dosing with quinine followed by primaquine. This led early investigators to conclude that quinine and chloroquine potentiated the radical curative effects of primaquine [140]. A pharmacokinetic interaction may contribute as chloroquine, piperaquine (a bisquinoline), and pyronaridine (a benzonaphthyridine derivative) increase plasma primaquine concentrations by 63%, 48% and 30%, respectively [74,141,142]. Whether this translates into increased clinical efficacy is unclear. Coadministration of mefloquine or artemether does not affect concentrations of primaquine [143,144].

#### 6.2.2. Challenges to primaquine use

##### 6.2.2.1. Adherence

Primaquine is generally recommended as a 14-day course for radical cure of *P. vivax* malaria, although there is a remarkable variety in national recommendations, despite limited evidence for the efficacy of alternative regimens. The optimum dose and duration is still debated. Most healthcare workers in countries endemic for *P. vivax* know that only a minority of patients who need primaquine actually receive it, regardless of what is recommended by treatment guidelines. Sometimes difficulty obtaining primaquine limits prescription [145], but more commonly the concern over potential hemolytic toxicity and also the requirement for adherence to a 14-day regimen influences the decision to treat. In migrant populations along the borders of Thailand, poor adherence has been well documented [146–148]. Similarly, in Peru, just over half the patients completed their primaquine regimens [149]. In contrast, high rates of adherence were reported in Afghanistan where 11/139 (8%) non-adherent individuals contributed 36 recurrences compared to 6/170 (4%) adherent individuals who contributed 19 recurrences [150]. This illustrates the higher burden of disease and higher transmission potential of non-adherent individuals. Studies of shorter course higher dose regimens are being conducted in several countries.

##### 6.2.2.2. Adverse effects

**Figure 5.**
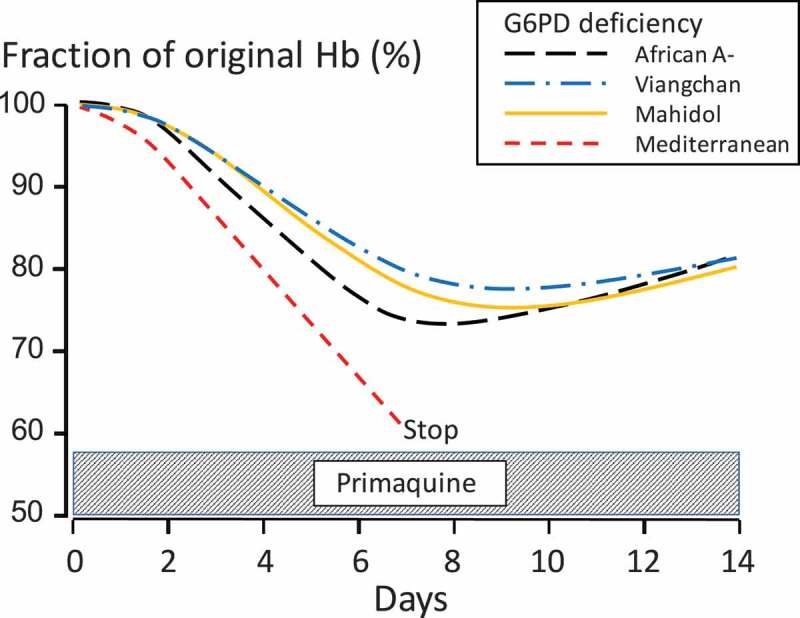
Hemolysis in G6PD deficiency during primaquine radical cure (30 mg/day in African and Mediterranean variants and 15 mg/day in Viangchan and Mahidol variants). After an initial delay of 1–2 days the average haemoglobin falls reaching a nadir 5–6 days after starting treatment, but then it rises again despite continued drug administration as young red cells with higher intraerythrocytic concentrations of G6PD enter the circulation. In the severe Mediterranean type of deficiency there is marked hemolysis and treatment must be stopped. Individual responses vary widely and so dangerous hemolysis can occur even in so called ‘mild deficiency’ genotypes. Figure available (open access): Ashley EA, Recht J, White NJ. Primaquine: the risks and the benefits. Malar J. 2014;13(1):418. (Full color available online)

Individual dosing of primaquine is limited by abdominal discomfort. This is often severe at doses over 1 mg base/kg. In general primaquine is well tolerated at individual doses ≤0.5 mg base/kg if given together with food [151,152]. Asymptomatic methemoglobinemia is common [153]. Symptoms develop when methemoglobin levels reach 10% of the normal level of hemoglobin. After dosing mean levels of methemoglobin usually range between 6% and 11%, but symptomatic disease has only been noted in patients with inborn deficiency of methemoglobin reductase [154]. The main adverse effect of primaquine is oxidant hemolysis [155,156]. Some red cell loss may occur in normal patients, but those who are G6PD deficient are particularly vulnerable. G6PD is necessary for the erythrocytes’ defenses against oxidant damage; it is required for the regeneration of reduced glutathione and the function of catalase. The erythrocytes of hemizygous G6PD-deficient males and homozygous females have less than 30% of the enzyme activity found in normal red cells (which, when measured at 30°C, is 7–10 IU/g of hemoglobin). There are over 180 different genetic G6PD variants, nearly all of which confer an unstable enzyme which degrades more rapidly than the normal variant and makes older red cells vulnerable to oxidant damage. The degree of hemolysis, and thus the risk, depends on two main factors; the degree of G6PD deficiency, and the dose and duration of exposure to primaquine [151,152]. Two of the most prevalent G6PD variants represent ends of the severity spectrum; the Mediterranean variant (the main variant found in Europe, West and Central Asia, and Northern India [157]) being among the most severe and the African A- variant (found in sub-Saharan Africa and in persons of African descent [157]) being among the mildest. There is also substantial variability in G6PD activity between individuals with the same genotype, and even within the same individual over time. Primaquine induced hemolysis in less severe G6PD variants typically starts after 1 or 2 days’ exposure when the older erythrocytes’ oxidant defenses have been depleted. If primaquine is continued in subjects with the African A-variant, or with the Viangchan or Mahidol mutations prevalent in SE Asia then hemolysis lessens, and the hemoglobin starts to rise again despite further drug administration as reticulocytes enter the circulation to replace the hemolyzed cells (Figure 5). These young red cells contain five times more G6PD than the oldest red cells and are relatively resistant to the hemolytic effects of primaquine. However, further hemolysis does occur with higher doses. In contrast, in the Mediterranean variant hemolysis continues if primaquine is not stopped, and life-threatening anemia may result. Only 15 deaths associated with primaquine have been reported over the past six decades [155,158] of which 13 were from severe hemolysis (one was due to hepatic necrosis and the cause of another was not stated). All but one death followed multiple doses [152]. In contrast hemolytic adverse effects but no deaths were reported from the mass treatments (MDA) in Jiangsu (>28 million treated) and from the combined experience in Azerbaijan, Afghanistan, Tajikistan, and DPR Korea (>8 million treated).

To reduce hemolytic toxicity in G6PD deficiency a weekly regime of (0.75 mg base/kg/week) for 8 weeks was devised nearly 50 years ago (see). This was evaluated only in the African A- G6PD variant [159–161]. Recently, in Cambodia, 18 G6PD-deficient male subjects (Viangchan variant) were given weekly primaquine. The maximum median fractional hemoglobin reduction from day zero was 16.3%, significantly greater than in G6PD normal subjects who had a reduction of 7.4% [162]. The safety of the weekly dose in different G6PD-deficiency genotypes requires more research.

##### 6.2.2.3. At risk and neglected populations

Medical supervision may be necessary to identify hemolysis especially in patients with multiple risk factors, i.e. medication history not revealed by the patient [162], illiteracy [163] or those with borderline severe anemia. Another important but neglected risk group are G6PD heterozygous females who commonly screen as ‘normal’ but, because their blood contains a mixture of normal and G6PD-deficient red blood cells, can theoretically hemolyze over half of their red blood cells. Primaquine is not recommended in pregnancy because the G6PD status of the fetus is not known. Because of uncertainty about excretion in breast milk primaquine is also not recommended in lactating women (unless the G6PD status of the baby is known). Due to lack of information rather than known adverse effects primaquine is also not recommended in infants less than 6 months of age. With these challenges, it can easily be understood why some health care providers are reluctant to prescribe primaquine, especially if they cannot check for G6PD deficiency.

#### 6.2.3. Radical curative efficacy of primaquine

The most widely used and studied primaquine dosing regimen has been 0.25 mg base/kg/day for 14 days (or 3.5 mg base/kg total dose). Studies evaluating this regimen in recent years originate from Thailand [113,114,148,164,165], Indonesia [121], India [166,167], Pakistan [168], Azerbaijan [169], Peru [170], Colombia [171,172], Brazil [110,173], and Ethiopia [174]. Before 2011, only 6 of 16 (38%) studies had follow-up durations greater than 28 days [110,113,164,171–173]. Studies with short follow up durations (28 days) measured asexual stage efficacy (recurrences were unusual) but if chloroquine was being evaluated they would have been very insensitive to low levels of chloroquine resistance. Longer follow up durations and use of control groups are necessary to measure the radical curative efficacy of primaquine. In the few studies with longer follow up, recurrence rates after primaquine (15 mg base/day for 14 days) were 4.6% at 3 months [113] and 17.5% at 6 months [164] in Thailand. Similarly, recurrence rates of 2.5% (dose 0.5 mg base/kg/day for 7 days) [173], 5% [173], and 14% [110] at 6 months were reported from Brazil (dose 15 mg base/day for 14 days) and 10.9–19% with 4–6 months follow up from Colombia (dose 0.25 mg base/kg/day for 14 days) [171,172]. To determine the efficacy of primaquine in endemic settings, assessment of recurrences is needed beyond the period of post treatment prophylaxis that follows blood stage treatment and for long enough to capture most relapses (ideally 6 months), while taking into account the incidence of new infections.

Since 2011, all seven studies assessing the radical curative efficacy of primaquine have had follow-up durations longer than 28 days, but they have not always included a control group so the assessment of efficacy has been relative. With a follow-up of 42 days the recurrence rates were 0% if chloroquine was the partner drug to primaquine (15 mg base/kg/day for 14 days in Thailand) [175]; 9% if artesunate–amodiaquine was the partner drug and 6% if dihydroartemisinin–piperaquine was the partner drug to primaquine (0.25 mg base/kg/day for 14 days in Indonesia) [121]. However, 6 weeks follow up is insufficient to detect all first relapses even in regions where the short-latency phenotype is prevalent. Studies with similar primaquine doses and longer follow up reported a recurrence rate of 0% at 3 months (Thailand) [148], and 8.1% at 6 months (compared to 16.4% without primaquine (India)) [167], and 13.6% (Peru) [176] with chloroquine as the partner drug. To detect relapses from long-latency phenotypes, follow-up durations should be longer than 9 months [177,178]. One year follow up following chloroquine plus primaquine allowed the detection of a 16.9% recurrence rate in India which was significantly less than a 26.7% recurrence rate in the control arm using chloroquine monotherapy [179]. In Peru, the recurrence rate 1 year after radical cure with primaquine 0.5 mg base/kg/day for 7 days was 29.4% [170]. In some areas of South East Asia and Oceania, high-dose primaquine (0.5 mg base/kg/day for 14 days) is used. Most studies measuring the efficacy of this dose used artesunate as the blood schizonticidal agent. Recurrence rates were <5% (Thailand) [116,117], 3.5% (Vietnam) [180], and in Papua New Guinea this regimen reduced the risk of recurrence by 28% [70]. Other high-dose primaquine studies have used sulfadoxine–pyrimethamine (Thailand) [118], dihydroartemisinin–piperaquine and quinine (Indonesia) [69], or primaquine as monotherapy (Thailand) [181]. One study evaluated high dose primaquine in combination with chloroquine; the recurrence rate was 1.8% over 11 months (Pakistan) [182]. In general, as the follow-up durations increase so do recurrence rates, suggesting that longer follow up is needed to capture all episodes. However, assessment is complicated because recurrences could either be due to new infections (longer follow-up obviously increases the probability of new infections too) or relapses (i.e. primaquine failure). These probabilities depend on latency phenotype (short or long), transmission intensity, relapse intervals, and the interval since treatment. Without accurate diagnostic tools to differentiate relapse from new infection, it is difficult to determine the true clinical efficacy of primaquine. Nonetheless, the combined evidence indicates that primaquine when given at a minimum total dose of 3.5 mg base/kg significantly reduces the risk of recurrence compared to when it is not given. With the increasing realization of the true burden of relapse, radical cure is now recommended in all transmission settings [183]. Most *P. vivax* endemic countries recommend a primaquine dosing regimen of 0.25 mg base/kg/day for 14 days, although there is a wide range of dosing regimens across countries [184] (Table 1).

**Table 1.** National recommendations for the treatment of *Plasmodium vivax* malaria between 2010 and 2015.

Country	2010	2011	2013	2015
Afghanistan	CQ only	CQ + PMQ-14	CQ + PMQ-8wk	CQ + PMQ-14 (0.25) or PMQ-8wk (0.75)
Argentina	ND	CQ + PMQ	CQ + PMQ-14 (0.25)	CQ + PMQ-14 (0.25)
Azerbaijan	CQ + PMQ-14	CQ + PMQ-14	CQ + PMQ-14 (0.25)	CQ + PMQ-14 (0.25)
Bangladesh	ND	CQ + PMQ-14	CQ + PMQ-14 (0.25)	CQ + PMQ-14 (0.25)
Belize	ND	CQ + PMQ	CQ + PMQ-14 (0.25)	CQ + PMQ-14 (0.25)
Bhutan	CQ + PMQ-14	CQ + PMQ-14	CQ + PMQ-14 (0.25)	CQ + PMQ-14 (0.25)
Bolivia	ND	CQ + PMQ	CQ + PMQ-7 (0.5)	CQ + PMQ-7 (0.5)
Brazil	ND	CQ + PMQ	CQ + PMQ-7 (0.5)	CQ + PMQ-7 (0.5)
Cambodia	ND	CQ only	DP only	DP + PMQ-14 (0.25)
China	ND	CQ + PMQ-8	CQ + PMQ-8	CQ + PMQ-8 (0.75)
Colombia	ND	CQ + PMQ	CQ + PMQ-14 (0.25)	CQ + PMQ-14 (0.25)
Costa Rica	ND	CQ + PMQ	CQ + PMQ-14 (0.25) or PMQ-7 (0.5)	CQ + PMQ-14 (0.25) or PMQ-7 (0.5)
DPR Korea	ND	CQ + PMQ-14	CQ + PMQ-14 (0.25)	CQ + PMQ-14 (0.25)
Domican Republic	ND	ND	CQ + PMQ-14 (0.25)	CQ + PMQ-14 (0.25)
El Salvador	ND	CQ + PMQ	CQ + PMQ-14 (0.25)	CQ + PMQ-14 (0.25)
Eritrea	CQ only	CQ + PMQ	CQ + PMQ	AS/AQ + PMQ-14 (0.25)
Ethiopia	CQ only	CQ only	CQ only	CQ only
French Guiana	ND	CQ + PMQ	CQ + PMQ	CQ + PMQ-14 (0.5)
Georgia	CQ + PMQ-14	CQ + PMQ-14	ND	ND
Guatemala	ND	CQ + PMQ	CQ + PMQ-14 (0.25)	CQ + PMQ-14 (0.25)
Guyana	ND	CQ + PMQ	CQ + PMQ-14 (0.25)	CQ + PMQ-14 (0.25)
Honduras	ND	CQ + PMQ	CQ + PMQ-14 (0.25)	CQ + PMQ-14 (0.25)
India	CQ + PMQ-14	CQ + PMQ-14	CQ + PMQ-14	CQ + PMQ-14 (0.25)
Indonesia	CQ + PMQ-14	AS/AQ or DP + PMQ-14	AS/AQ or DP + PMQ14 (0.25)	AS/AQ or DP + PMQ-14 (0.25)
Iran	ND	CQ + PMQ-14	CQ + PMQ-14 or PMQ-8wk (0.75)	CQ + PMQ-14 or PMQ-8wk (0.75)
Iraq	ND	CQ + PMQ-14	ND	ND
Kyrgyzstan	ND	CQ + PMQ-14	CQ + PMQ-14 (0.25)	ND
Lao PDR	CQ + PMQ-14	CQ + PMQ-14	CQ + PMQ-14	CQ + PMQ-14
Malaysia	CQ + PMQ-14	CQ + PMQ-14	CQ + PMQ-14 (0.25)	CQ + PMQ-14 (0.5)
Mexico	ND	CQ + PMQ	CQ + PMQ	CQ + PMQ-14 (0.25)
Myanmar	ND	CQ + PMQ-14	CQ + PMQ-14 (0.25)	CQ + PMQ-14 (0.25)
Nepal	ND	CQ + PMQ-14	CQ + PMQ-14	CQ + PMQ14 (3.75 to 15 mg/day)
Nicaragua	ND	CQ + PMQ	CQ + PMQ-7 (0.5)	CQ + PMQ-7 (0.5)
Pakistan	ND	CQ + PMQ-14	CQ + PMQ-14 (0.25)	CQ + PMQ-14 (0.25)
Panama	ND	CQ + PMQ	CQ + PMQ-7 or PMQ-14 (0.25)	CQ + PMQ-7 or PMQ-14 (0.25)
Papua New Guinea	ND	AL + PMQ	AL + PMQ	AL or QN + PMQ-14 (7.5 mg/day)
Paraguay	ND	CQ + PMQ	CQ + PMQ-14 (0.25)	CQ + PMQ-14 (0.25)
Peru	ND	CQ + PMQ	CQ + PMQ	CQ + PMQ-7 (0.5)
Philippines	ND	CQ + PMQ-14	CQ + PMQ-14 (0.25)	CQ + PMQ-7 (0.5)
Republic of Korea	ND	ND	CQ + PMQ-14 (0.25)	CQ + PMQ-14 (0.25)
Saudi Arabia	CQ + PMQ-14	CQ + PMQ-14	CQ + PMQ-14	CQ + PMQ-14 (0.25)
Solomon Islands	ND	CQ + PMQ-14	AL + PMQ-14 (0.25)	AL + PMQ-14 (0.25)
Somalia	ND	CQ + PMQ-14	CQ + PMQ-14	NR
South Africa	ND	AL or CQ + PMQ	AL or CQ + PMQ	AL or CQ + PMQ
South Sudan	ND	CQ only	AS/AQ + PMQ	AS/AQ + PMQ
Sri Lanka	CQ + PMQ-14	CQ + PMQ-14	CQ + PMQ-14 (0.25)	CQ + PMQ-14 (0.25)
Sudan (north)	ND	AL only	AL + PMQ-14 (0.25)	AL + PMQ-14 (0.25)
Suriname	CQ + PMQ	CQ + PMQ	CQ + PMQ-14 (0.25)	CQ + PMQ-14 (0.25)
Tajikistan	ND	CQ + PMQ-14	CQ + PMQ-14 (0.25)	CQ + PMQ-14 (0.25)
Thailand	ND	CQ + PMQ-14	CQ + PMQ-14 (0.25)	CQ + PMQ-14 (0.25)
Timor-Leste	ND	CQ + PMQ-14	CQ + PMQ-14 (0.5)	CQ + PMQ-14
Turkmenistan	ND	ND	ND	ND
Uzbekistan	ND	CQ + PMQ-14	CQ + PMQ-14 (0.25)	ND
Vanuatu	ND	CQ + PMQ-14	AL + PMQ-14 (0.25)	AL + PMQ-14 (0.25)
Venezuela	ND	CQ + PMQ	CQ + PMQ-14 (0.25)	CQ + PMQ-14 (0.25)
Vietnam	ND	CQ + PMQ-14	CQ + PMQ-14 (0.25)	CQ + PMQ-14 (0.25) or (15 mg/day)
Yemen	ND	CQ + PMQ-14	CQ + PMQ-14 (0.25)	CQ + PMQ-14 (0.25)

Data compiled from the World Health Organization, World Malaria Reports accessed between Feb-Mar 2016, open access: http://www.who.int/malaria/publications/country-profiles/en/I. Only countries endemic for *P. vivax* with >1 year of data are included. In parenthesis is the daily mg base/kg dose unless otherwise indicated. Abbreviations: ND: no data; NR: not reported; CQ: chloroquine, PMQ: primaquine; DP: dihydroartemisinin–piperaquine; AL: artemether–lumefantrine; AS/AQ: artesunate–amodiaquine; PMQ-14: 14-day course of primaquine; PMQ-7: 7 day course of primaquine; PMQ-8wk: weekly for 8 weeks primaquine; PMQ-8: 8 day course of primaquine.

#### 6.2.4. Radical cure and G6PD deficiency

In G6PD-deficient persons, primaquine given weekly for 8 weeks is recommended for radical cure. In 1960, a comparison of weekly primaquine regimens for radical cure in males with presumed African A-G6PD deficiency found the efficacy of 45 mg weekly for 8 weeks to be less than that of the 60 mg weekly regimen; 10% failure versus 6%, respectively (follow-up duration not reported) [160]. Based on the results of this study as well as a previous study on 30 mg weekly for causal prophylaxis [159], the authors concluded that ‘considering the safety gained by the use of 45 mg, and the relatively slight gain in therapeutic effect that is obtained by the higher dose of 60 mg base per week, the lower dose would seem preferable for field use’. Since 1960, there have been few studies on weekly primaquine for radical cure. In Iran, a weekly dose of 0.75 mg base/kg for 8 weeks resulted in a failure rate of 4.6% at 1 year follow up [185]. Three years later in 2008, equivalent failure rates were reported in Pakistan; 1.8% for primaquine 0.5 mg/kg/day for 14 days and 5.1% for 0.75 mg/kg weekly for 8 weeks as compared to 31% for placebo [182]. A subsequent study in Cambodia demonstrated that the 0.75 mg/kg weekly primaquine dose was well tolerated, however, one G6PD-deficient male (with additional hemolytic risk factors) required blood transfusion [162].

#### 6.2.5. Primaquine ‘resistance’

Primaquine tolerance or resistance in *P. vivax* has been an area of uncertainty and confusion [88,186–189]. With fixed doses, overweight patients may be underdosed [190], and this can be mistakenly attributed to primaquine resistance. Recent evidence has shown that the conversion of primaquine to its bioactive metabolites relies on the liver cytochrome P450 CYP isoform CYP2D6 [133,191]. Poor or intermediate metabolizers may have lower therapeutic activity and increased risk of relapse. ‘Resistance’ in this case would have a pharmacokinetic basis. Studies measuring primaquine efficacy will require concomitant pharmacogenetic or phenotypic assessments [191,192] to determine whether or not primaquine ‘resistance’ exists.

#### 6.2.6. Alternative treatments to primaquine

Tafenoquine, a slowly eliminated 8-aminoquinoline structurally related to primaquine was first tested for safety and tolerability in G6PD normal males in 1998 [193]. Early studies confirmed the anti-relapse efficacy of tafenoquine [194,195]. More recently, a multi-center phase 3 trial (DETECTIVE) across seven sites in Brazil, Peru, India, and Thailand showed that a single 300 or 600 mg dose of tafenoquine in combination with chloroquine was as efficacious as primaquine 15 mg/kg/day for 14 days in preventing *P. vivax* relapse [196]. Whether tafenoquine is critically reliant upon CYP2D6 for metabolism into active metabolite(s) is not yet clear with contradictory results from animal [197] and human studies; in clinical trials of tafenoquine, decreased CYP2D6 activity was not associated with an increased risk of *P. vivax* relapses [198]. Tafenoquine, as with primaquine, causes hemolysis in G6PD deficiency. However in contrast to the slowly eliminated tafenoquine, the rapidly eliminated primaquine can be stopped if there is serious hemolysis. Thus, the greatest advantage and disadvantage of tafenoquine lies with its single dose regimen. The deployment of tafenoquine will likely be accompanied by improved methods of G6PD testing.

## 7. Assessing the efficacy of *P.* *vivax* treatments

The blood stage efficacy of slowly eliminated antimalarials (i.e. chloroquine, mefloquine, piperaquine) can be assessed within 28 days. This assesses both the activity of the drug against the initial infection and its ability to suppress relapse parasites emerging from liver hypnozoites 2 weeks after starting treatment [199]. For rapidly eliminated drugs (i.e. artemisinins alone, quinine) early relapses are not prevented so low grade resistance is difficult to detect. Thus comparing the blood stage activity of two drugs with different elimination profiles may lead to erroneous conclusions. For example with short-latency *P. vivax*, lumefantrine does not suppress recurrences after approximately 1 month whereas piperaquine does. Thus at 6 weeks, lumefantrine will have a higher ‘failure’ rate compared to piperaquine.

There are at least five different primaquine regimens recommended by different national guidelines (Table 1) and even more primaquine regimens that have undergone efficacy studies in combination with other antimalarials. Radical curative activity should be assessed in combination with effective asexual stage drugs to minimize the risk of confounding recrudescences, and recurrence rates should be compared over a period sufficient to capture most initial relapses. Six months’ follow up is ideal in tropical areas as this will capture nearly all relapses, but shorter periods (i.e. 3–4 months) will still capture the majority. As relapses can be homologous or heterologous to the primary infection, there are no methods to distinguish reliably relapse from reinfection (when the recurrence is of a different genotype); and after 14 days recrudescence also cannot be reliably distinguished (if the recurrence is of a similar genotype). This confounds the interpretation of studies conducted in endemic settings. Reinfection can be excluded in studies outside endemic areas but these usually involve travelers or soldiers who may not represent the local population at risk and thus may have different recurrence patterns. Nevertheless, comparing total recurrence rates with two or more regimens is still informative and can be used to guide treatment policy.

## 8. Vaccines for *P.* *vivax* malaria

Development of a vaccine for *P. vivax* lags behind *P. falciparum*. Potential targets identified include *P. vivax* circumsporozoite protein (*Pv*CSP), *P. vivax* merozoite surface protein (*Pv*MSP-1_19_), *Pv*MSP-9_NT_, and *P. vivax* apical membrane antigen (*Pv*AMA1) [200]. Several vaccine candidates are currently in, or have completed phase 1 testing [201,202]. An efficacious vaccine protective against *P. vivax* may not be available generally for years to come.

## 9. Conclusion

The treatment for the blood stage of *P. vivax* infections is chloroquine (25 mg base divided over 3 days) or an ACT (except one containing sulfadoxine–pyrimethamine), such as artesunate–mefloquine, artemether–lumefantrine, artesunate–pyronaridine, or dihydroartemisinin–piperaquine. If radical treatment with primaquine is not given, then the more slowly eliminated ACTs are preferred to artemether–lumefantrine as they delay and may prevent relapses. However, there is increasing acceptance that radical treatment to prevent relapse should be given in all endemic settings. Improving radical cure is the greatest therapeutic challenge in vivax malaria. When G6PD status is normal by screening tests, the standard primaquine dose is 0.25 mg base/kg/day for 14 days (total dose 3.5 mg base/kg) except in East Asia and Oceania where a higher dose 0.5 mg base/kg/day (total dose 7 mg base/kg) for 14 days is recommended. Whether the duration of primaquine can be shortened (providing the same total dose, and thus higher daily dosing) is under investigation. For G6PD-deficient patients with ready access to medical facilities, primaquine 0.75 mg base/kg once weekly for 8 weeks is recommended only if the local G6PD variants are known to be of mild or moderate severity. Patients should be advised to stop taking primaquine and seek medical attention if they pass black urine. Single dose tafenoquine is a promising simpler alternative to the 14 day primaquine course, but the potential for prolonged hemolysis in G6PD-deficient individuals means that sensitive testing must accompany its deployment. Vaccines and the development of non-hemolytic drugs against hypnozoites are hopes for the future.

## 10. Expert commentary

*P. vivax* is an important cause of morbidity in many parts of the tropical world, although it is rare in most of Africa (except for the horn and northwest Africa). Compared to *P. falciparum, P. vivax* is less likely to cause severe malaria, although it may kill directly in high transmission settings where repeated infections cause severe anemia in young children, or indirectly through reduction in birth weight and consequent increased infant mortality. Symptomatic vivax malaria is diagnosed by blood smear or a rapid diagnostic test (RDT). RDTs that detect *P. vivax* are a valuable addition to malaria control activities but are slightly less sensitive than the corresponding tests for *P. falciparum*. Chloroquine, the standard treatment against the blood stage of *P. vivax* for the past 60 years has generally remained effective, but resistance has now been reported in over 10 countries and high grade resistance is prevalent in Indonesia and Papua New Guinea. Artemisinin combination treatments are now recommended as alternative first line treatments, allowing a unified approach to the treatment of all malaria. The main therapeutic challenge in vivax malaria is prevention of relapse. The only currently recommended treatment that eradicates the hypnozoites which cause relapse (‘radical cure’) is primaquine. There is no evidence of acquired resistance to primaquine. Because primaquine causes hemolysis in patients who have G6PD deficiency, and testing for G6PD deficiency is usually unavailable, primaquine is often not prescribed. When it is prescribed the effectiveness of radical cure depends on adherence to the 14-day treatment regimen. Assessment of the risks and benefits of radical cure is challenged by the variability in the prevalence and severity of G6PD deficiency (over 180 genetic variants are described), and substantial differences in the frequency and pattern of relapse between different geographic areas.

## 11. Five-year view

If the world makes continued progress towards elimination of *P. falciparum*, greater attention will be needed to the approaches required to eliminate the other malarias, of which by far the most important is *P. vivax*. Elimination can proceed slowly, as it did once in Europe, North America and the USSR by continued strengthening of control activities and economic development, or more rapidly by trying to eliminate all malaria parasites in the human population (notably those in individuals who are clinically well) in malaria endemic areas. Both approaches are challenged by insecticide resistance in anopheline vectors and drug resistance in malaria parasites. New antimalarial treatments will be introduced for *P. falciparum* malaria and these will need to be evaluated also in *P. vivax* malaria. Reliable rapid diagnostic tests for G6PD deficiency are being developed and these can promote wider use of radical treatment. The new generation of G6PD deficiency tests will be quantitative allowing identification of heterozygous females who have a hemolytic risk but are usually reported as ‘normal’ with currently available rapid G6PD deficiency tests. Individual variations in CYP2D6 activity affect the efficacy of primaquine, and it is likely that pharmacogenetic profiling will become more readily available. In the next 5 year the safety and effectiveness of shorter courses of primaquine will have been established, perhaps providing safer regimens, and alternative regimens in patients with G6PD deficiency will have been investigated. Research to develop safer drugs for radical cure will continue. The only advance likely in the next 5 years is the deployment of tafenoquine providing single dose radical cure – but also potential for greater hemolysis in G6PD deficiency will necessitate concomitant use of a more sensitive test for G6PD deficiency. Mass treatment approaches have a long and chequered history but will likely be needed for rapid elimination and will be facilitated by the availability of tafenoquine. Candidate vivax malaria vaccines have been developed but are unlikely to become generally available within 5 years.

## Key issues

- In most *P. vivax* endemic countries, chloroquine is still efficacious against the blood stage of *P. vivax* malaria but resistance is increasing.
- All ACTs, except those containing sulfadoxine-pyrimethamine, can be used as first line treatments for the treatment of blood stage *P. vivax* infection.
- Relapse is a major contributor to vivax malaria illness. In East Asia and Oceania repeated relapses of *P. vivax* malaria cause substantial morbidity and contribute to mortality from anaemia in childhood.
- Relapse patterns vary geographically.
- Differentiating *P. vivax* recrudescence from relapse and re-infection remains a significant challenge.
- Radical cure of vivax malaria must be given more widely.
- Radical cure requires use of 8-aminoquinoline drugs, of which primaquine is the only widely available agent. These drugs all cause hemolysis in patients with G6PD deficiency.
- Potential alternatives to the standard 14-day course of primaquine under investigation are single dose tafenoquine or shorter higher dose courses of primaquine.
- Recommended treatment for radical cure in G6PD normal patients is primaquine 0.25 mg base/kg/day for 14 days (total dose 3.5 mg base/kg) but in East Asia and Oceania a higher dose is recommended: 0.5 mg base/kg/day (total dose 7 mg base/kg) for 14 days.
- When G6PD deficient variants are of mild to moderate severity, recommended radical curative treatment for G6PD deficient patients is primaquine 0.75 mg base/kg/wk for 8 weeks.
